# Soil Yeast Communities in Revegetated Post-Mining and Adjacent Native Areas in Central Brazil

**DOI:** 10.3390/microorganisms8081116

**Published:** 2020-07-24

**Authors:** Geisianny Augusta Monteiro Moreira, Helson Mario Martins do Vale

**Affiliations:** 1 Microbial Biology Graduate Program, Biological Sciences Institute, Universidade de Brasília, Campus Darcy Ribeiro, Asa Norte, 70910-900 Brasília/DF, Brazil; gamm.bio@gmail.com; 2 Laboratory of Mycology, Department of Phytopathology, Biological Sciences Institute, Universidade de Brasília, Campus Darcy Ribeiro, Asa Norte, 70910-900 Brasília/DF, Brazil

**Keywords:** Ascomycetous yeasts, *Candida* species, phytocenoses, iron-mining, seasonality, soil ecology, tropical soils

## Abstract

Yeasts represent an important component of the soil microbiome. In central Brazil, mining activities are among the main anthropogenic factors that influence the dynamics of the soil microbiota. Few studies have been dedicated to analysis of tropical soil yeast communities, and even fewer have focused on Brazilian hotspots influenced by mining activity. The aim of the current study was to describe soil yeast communities in a post-mining site with revegetated and native areas, along Neotropical Savanna and Atlantic Forest biomes. Yeast communities were described using a culture-based method and estimator-based species accumulation curves, and their associations with environmental characteristics were assessed using multivariate analysis. The results indicate a greater species richness for yeast communities in the revegetated area. We identified 37 species describing 86% of the estimated richness according to Chao2. Ascomycetous yeasts dominated over basidiomycetous species. *Candida maltosa* was the most frequent species in two phytocenoses. Red-pigmented yeasts were frequent only in the summer. The main soil attributes affecting yeast communities were texture and micronutrients. In conclusion, each phytocenosis showed a particular assemblage of species as a result of local environmental phenomena. The species richness in a Revegetated area points to a possible ecological role of yeast species in environmental recovery. This study provided the first comprehensive inventory of soil yeasts in major phytocenoses in Minas Gerais, Brazil.

## 1. Introduction

Yeasts are a taxonomically heterogeneous group of predominantly unicellular fungi [1]. Yeast communities in soil are diverse and globally distributed [2] and are influenced by a variety of environmental factors [3,4,5,6]. In Brazil, studies on soil yeasts are still few, despite the diversity of vegetation types present, including recognized hotspots of global diversity, such as Brazilian Atlantic Forest and Neotropical Savanna. The first report on yeasts in Brazilian soils described the ecology of pathogenic yeasts in Amazonian soils [7]. *Candida* species were the most frequent, and several species of the genera *Debaryomyces*, *Cryptococcus*, and *Rhodotorula* were also encountered. Yeast communities associated with sugarcane rhizospheres during different phases of plant development were studied in Rio de Janeiro [8], demonstrating a higher portion of basidiomycetous than ascomycetous isolates. In 2002, mycocinogenic yeasts were isolated from Amazon rain forest soils, with a predominance of ascomycetous species [9]. In 2003, the presence of basidiomycetous yeasts in the corn rhizosphere was reported in senescent plants [10]. The first extensive inventory of yeasts isolated from Neotropical Savanna soils in Minas Gerais was carried out in 2013, including geographical and seasonal distribution, and enzyme production [11]. In Brazil, an effort is still required to fill the knowledge gaps regarding the diversity of yeasts in soil and the factors that influence communities. Among the factors that can influence soil yeast communities in central Brazil, human activities such as mining have been insufficiently explored.

The state of Minas Gerais, in central Brazil, has strong ties to mining activity, and iron mining is the basis of the economy of several municipalities. Mining activities in general account for 5 to 6% of Brazil’s total gross domestic product [12,13]. The state of Minas Gerais has significant mineral deposits concentrated mainly in the region known as the Iron Quadrangle (*Quadrilátero Ferrífero*), which occupies about 7000 km^2^ of the southeastern portion of the state. The region has a great species richness of vascular plants, with its vegetation cover shared between the Neotropical Savanna and Brazilian Atlantic Forest biomes. The Brazilian Atlantic forest biome is currently very degraded, being mainly represented by fragments of vegetation. The Neotropical Savanna is composed of a mosaic of phytophysiognomies, ranging from forest areas to open areas with very sparse vegetation. The prevailing hot climate in the Iron Quadrangle (Cwa—humid temperate, according to Köppen’s classification), has an average annual temperature of 20 °C, and precipitation ranging from 1300 to 2100 mm per year. The region has two well-defined seasons: dry winter and rainy summer [14].

Despite its economic importance, iron mining causes an environmental impact on soil structure with consequent reduction of organic matter and nutrients, resulting in modifications to the native vegetation and soil microbiota [15]. Recovery of mining-modified areas is critical to reestablishing biological processes within the affected ecosystem [16]. The recovery of degraded areas is usually achieved by establishing the plant community, which depends on a diverse and functional soil microbial community [17]. Brazil has legal norms that define the restoration of degraded ecosystems as closely as possible to natural conditions, or rehabilitation that restores such regions to a non-degraded state with a natural balance, which may be different from natural conditions [16]. The legal norms specify criteria for the selection of plant species, prioritizing native and autochthonous species, where non-native and non-invasive species are used only in justified cases [16]. For example, restoration with tree species in a post-bauxite mining area has provided ecological benefits, such as reduced erosion and reduced invasion by aggressive exotic grasses [18]. Some biological indicators demonstrate the successful restoration of the plant community, with similarity to reference systems in terms of taxonomic, functional, and phylogenetic diversity of plants, soil microbial communities and pollinator communities [19]. 

A great effort has been made to describe soil microbiota diversity in the Iron Quadrangle region in order to compare natural and disturbed ecosystems at iron mining sites. Using the same set of soil samples, several functional groups of soil microorganisms have been studied. Among common observations, diversity indexes were higher in disturbed ecosystems at iron mining sites than in natural ecosystems. The bacterial community was more diverse at an environmental recovery mining site. Proteobacteria represented the predominant phylum [20], and a greater genetic diversity of nitrogen-fixing bacteria was found in the rehabilitated area that had been revegetated with grass [21]. Arbuscular mycorrhizal fungal communities were more diverse in revegetated sites than in the adjacent original ecosystems [13,22]. The soil microbiome showed the highest values of alpha and beta diversity in revegetated sites [23]. To our knowledge there has been only one study in Brazil investigating soil yeasts in natural and disturbed ecosystems in the same iron-mining site mentioned above [24]. The yeast community in this area was composed of six genera, and *Saitozyma podzolica*, a typical pedobiont species, was the most common species. As noted for the other soil functional groups [13,20,21,22], yeast communities were more diverse in disturbed ecosystems at iron mining sites than in natural ecosystems. These reports provide evidence on the role of soil yeasts, with other soil functional groups, in the recovery of degraded lands.

Due to the lack of attention given to this group in Brazilian soil ecosystems, we aimed to: (a) inventory the yeast species in different phytocenoses of post-mining sites along the Neotropical savanna and Atlantic forest biomes in Minas Gerais, Brazil, (b) evaluate the yeast community’s dynamics across the seasons, and (c) characterize the relationship between the yeast communities, the vegetation type, and the basic soil physicochemical properties. We hypothesized that modifying the vegetation at the post-mining site would alter the composition of yeast communities with an increase in species richness.

## 2. Materials and Methods

### 2.1. Study Area and Sampling Design

The study areas were located near the cities of Brumadinho and Nova Lima, both within the Iron Quadrangle region, in the Minas Gerais state, Brazil. The two areas feature iron-mining sites, known as the *Córrego do Feijão* (Brumadinho) and *Miguelão* (Nova Lima) mine areas, belonging to the Vale SA Company. This region has a warm temperate climate with two well-defined seasons: dry winter (May to September) and rainy summer (October to April). The mean annual temperature and precipitation are 21 °C, and 1300 mm per year (Table 1). Soil samples were collected in four phytocenoses: Atlantic forest (AF), Iron outcrops (IO), Neotropical savanna (NS), and Revegetated (RA) areas.

**Figure 1:**
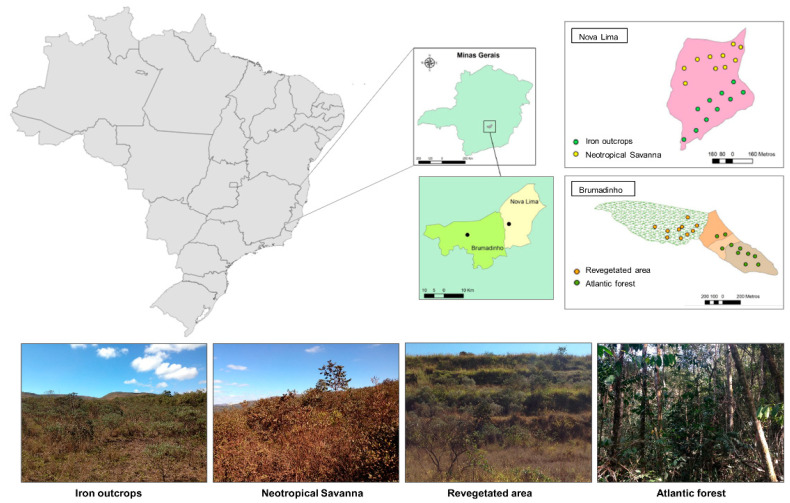
Geographical location of studied areas, sampling points, and phytocenoses.

Atlantic forest constitutes a seasonal semi-deciduous montane forest, part of the Brazilian Atlantic Forest, with a variety of tree species. Neotropical savanna is a savanna type of vegetation with a predominance of grasses, small trees, herbaceous, and shrubs, that are quite sparsely distributed, with trees generally isolated. Iron outcrops constitute an ecotone between Neotropical savanna and Atlantic forest, associated with mountain plateaux and superficial iron crusts where herbs, grasses, and shrubs predominate, and there may be underdeveloped trees. This ecosystem has a high rate of endemic plant species and is among the most threatened and least studied in Minas Gerais state. The Revegetated area constitutes an iron-mining site under process of rehabilitation. It is formed from slopes made with sterile tailings to stabilize the iron ore storage area (Figure 1). The rehabilitation process in the Revegetated area began in 2005, when mining activity ceased. Currently the area is dominated by grasses such as *Urochloa brizantha* and *Panicum maximum*.

The dominant soil type in the studied phytocenoses, according to the World Reference Base for Soil Resources [25], was as follows: Ferralsols and Cambisols in Brumadinho, and Plinthosols in Nova Lima. The Revegetated area soils were classified as an anthropogenic terrain. Further description of the soil characteristics has been published previously [26].

Soil samples were collected in the dry winter (August 2015) and rainy summer (January 2016). For each area, two transects were delimited, 50 to 70 m apart from each other. In each transect, five composite samples were collected (0–0.2 m depth), resulting in 10 soil samples per area in each season. A total of 80 soil samples were collected (4 sites, 2 seasons, and 10 samples) (Figure 1). Samples were placed in plastic bags and stored at 4 °C to be analyzed at the Mycology laboratory of the Department of Plant Pathology at the University of Brasília—UnB, Brasília, Brazil.

Soil chemical analyses were carried out at the Federal University of Lavras—UFLA, Brazil, according guidelines from *Empresa Brasileira de Pesquisa Agropecuária*—EMBRAPA [27]. Concentrations of phosphorus (P), potassium (K), calcium (Ca), magnesium (Mg), aluminum (Al), zinc (Zn), iron (Fe), manganese (Mn), copper (Cu), boron (B), sulfur (S), exchangeable acidity (H + Al), organic matter (OM), remaining phosphorus (P-rem), sand, silt, and clay were evaluated. These results were used to calculate other parameters, such as the sum of exchangeable bases (SB), cation exchange capacity at pH 7.0 (T), effective cation exchange capacity (t), aluminum saturation index (m), and base saturation index (V). The pH of all samples was also determined.

### 2.2. Yeast Isolation and Identification

For the isolation of cultivable yeasts, aliquots of 10 g of soil were homogenized with 50 mL of YM broth (0.3% Malt extract; 0.3% Yeast extract; 0.5% Peptone; 1% Glucose), and stirred for 16 h at 120 rpm and 28 °C. All soil samples were analyzed in three replicates, and each of the replicates was used to produce three dilutions (10^−1^, 10^−2^, and 10^−3^). The dilutions were plated on YM agar (0.3% Malt extract; 0.3% Yeast extract; 0.5% Peptone; 1% Glucose; 2% Agar). Sodium propionate (0.25%) and chloramphenicol (100 µg mL^−1^) were used to inhibit the growth of filamentous fungi and bacteria, respectively. Incubation of all plates was performed at room temperature until the growth of colonies (minimum of three days). All yeast colonies grown were selected and purified on YM agar, and differentiated into macro morphological types (color, aspect, margin) using a dissection microscope [28]. Pure cultures were cryopreserved (−80 °C) using YM broth with 25% glycerol.

DNA extraction was performed from the cell precipitate, obtained by centrifugation of the yeast culture grown in YM broth over 48 h at 28 °C under stirring, as previously described [24]. The D1/D2 domains of the ribosomal LSU (Large Ribosomal Subunit) gene were amplified by PCR, using the following universal primers: NL1 (5′-GCA TAT CAA TAA GCG GAG GAA AAG-3′) and NL4 (5′-GGT CCG TGT TTC AAG ACG G-3′) [29,30]. The PCR conditions were as follows: initial denaturation at 94 °C for 3 min, followed by 33 cycles of denaturation at 94 °C for 1 min, annealing at 56 °C for 30 s, and extension at 72 °C for a 1 min, and final extension at 72 °C for 6 min. The PCR products were treated with the Exo-SAP enzyme (Affymetrix, Inc. Cleveland, Ohio, USA) and sent for sequencing at ACTGene Molecular Analyses (Rio Grande do Sul, Brazil), using the sequencer ABI-Prism 3500 Genetic Analyzer (Applied Biosystems). For species identification, the obtained nucleotide sequences were compared with sequences deposited in the NCBI GenBank Database (www.ncbi.nih.gov), using the BLASTn algorithm [31]. The sequences of the isolates were deposited in GenBank, with the accession codes: MK110049 to MK110345.

### 2.3. Community Structure and Multivariate Statistics

Multivariate and community structure analyses were performed with incidence-based data (presence/absence), based on species occurrence frequency observed in the samples. The occurrence frequency was calculated as a proportion of the number of isolates of each species divided by the total number of isolates observed in each sample. Estimator-based species accumulation curves were calculated with EstimateS 9.1 [32], using default settings. Two estimators of species richness were used: Chao2 richness estimator and ICE incidence-based coverage estimator. The ICE estimator distinguishes between rare and frequent species, while Chao2 is based on the incidence of species richness [33].

Nonmetric multidimensional scale (NMDS) was used to visualize the differences between the phytocenoses, soil physicochemical properties, and the seasonal dynamics in relation to yeast community composition. NMDS is a rank-based approach, which produces an ordination based on a distance or dissimilarity matrix. The Jaccard index was used and an R test statistic (stress values) [34]. Among the soil variables (e.g., nutrient content, organic matter), those having the most significant influence on the microbial community structure were chosen by forward selection with a permutation test. The NMDS was performed with the Vegan package [35]. All the analysis was done with the R software [36].

## 3. Results

### 3.1. Soil Characterization

Soil chemical characterization (Table 2) was performed using the recommendations of the Soil Fertility Commission of the Minas Gerais state, Brazil [37]. Soils ranged from medium acidity (Revegetated area) to high acidity (Neotropical savanna, Iron outcrops, and Atlantic forest areas). Considering the content of organic matter (OM), and the cation exchange complex (Ca^2+^, Mg^2+^, Al^3+^, SB, H+Al, t, T, and m), as parameters related to soil fertility, all areas presented low fertility conditions, with the Revegetated area being the one with the lowest values. The Revegetated area also showed the highest values for base saturation (V), probably due to the revegetation process. Atlantic forest soils had the highest clay content, and the Iron outcrops soils had the highest sand content.

The Zn content was within the limits established for soil fertility, ranging from medium (Atlantic forest, Revegetated areas) to good (Neotropical savanna, Iron outcrops areas). Mn, Fe, and S presented the highest concentrations in the soil samples, while Iron outcrops area had the highest Fe content. Cu and B did not exceed the maximum allowed concentrations established by Brazilian environmental regulations for soil (CONAMA). The great majority of the soil attributes showed a statistically significant difference between the evaluated phytocenoses. In terms of soil physicochemical characterization, the Revegetated area was the most distinct. The soil physicochemical parameters were not significantly different between sampling seasons, as they followed the same tendency during the dry winter and rainy summer.

### 3.2. Yeast Diversity

**Figure 2:**
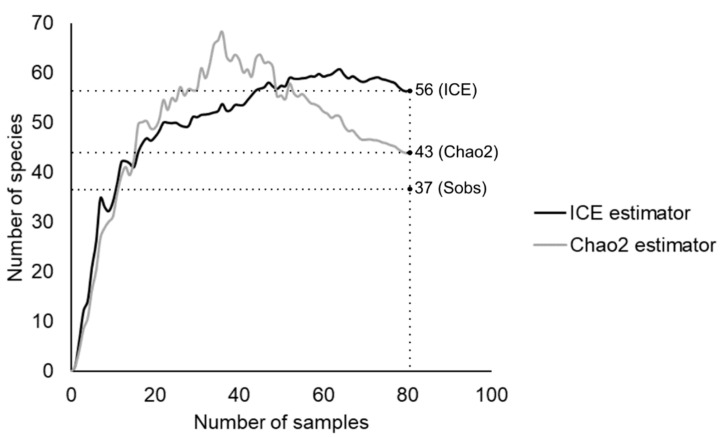
Estimator-based species accumulation curves obtained with the incidence-based coverage (ICE) and Chao2 estimators for total set samples. Sobs represents the species richness observed in the data set.

A total of 295 isolates were obtained in the present study. Cultivable yeasts were detected in 57 of the 80 soil samples evaluated. The total observed richness was 37 taxa, and total richness estimation for all sites indicated by the Chao2 and ICE estimators were 43 and 56 taxa, respectively (Figure 2). The sample effort was sufficient to describe 86 and 66% of the richness estimation by Chao2 and ICE estimators respectively, in the study area.

A high number of ascomycetous species were observed (29 taxa), with all species belonging to the order Saccharomycetales. In the Basidiomycota, eight species were observed, belonging to the orders Tremellales and Sporidiobolales (Table 3). Most of the species found represented less than 10% of occurrence frequency, and nine species were represented by a single isolate (singletons) in all samples. For *Candida* sp. the LSU gene was not sufficient to distinguish at the species level. The isolates of the genera *Schwanniomyces* sp., *Debaryomyces* sp., *Meyerozyma* sp., and *Torulaspora* sp. represent possible new species and are already being further studied by our research team. For some ascomycetes, in addition to sequencing the ITS region, sequencing of protein-coding genes, e.g., ACT for *Debaryomyces* and *Schwanniomyces*, TEF1 for *Hanseniaspora*, will be required. Sequencing and analysis of other marker genes are being conducted for confirmation.

### 3.3. Yeast Communities by Phytocenoses

The structure of yeast communities diverged between phytocenoses. The Revegetated area showed the highest species richness (20 taxa), followed by Atlantic forest area with 17 taxa. The Iron outcrops area presented 13 taxa, and the Neotropical savanna area had 9, presenting the lowest species richness among phytocenoses (Table 3). In the Revegetated area, the most frequent species were *Candida maltosa* (60%), *Papiliotrema laurentii* (11%) and *Rhodotorula mucilaginosa* (6%). The other species showed a less than 5% frequency of occurrence, and nine species represented singletons. *Candida maltosa* was also the most frequent species detected in the Atlantic forest area (31%), followed by *Schwanniomyces vanrijiae* (25%) and *Saitozyma podzolica* (8%). In the Iron outcrops area, the most frequent species was *Candida melibiosica* (43%), and *Candida intermedia* (20%) in the Neotropical savanna area.

**Figure 3:**
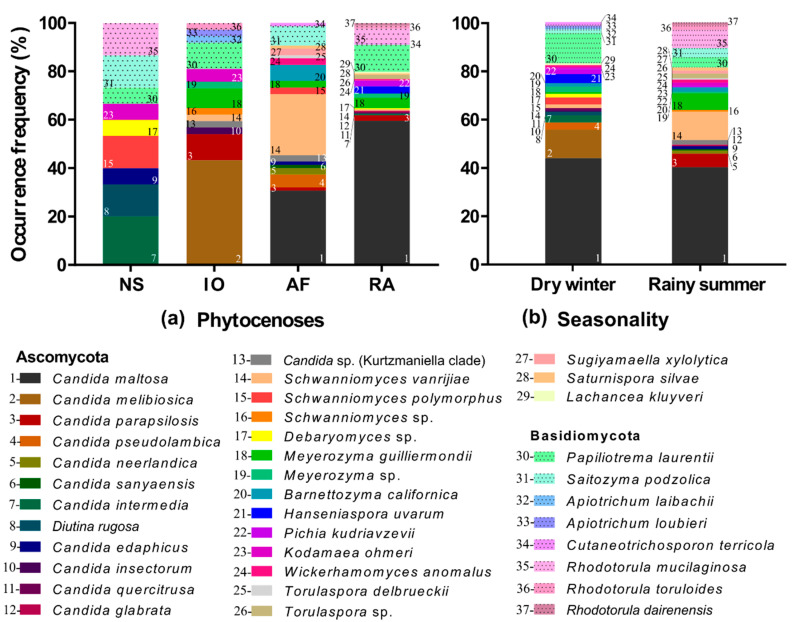
Yeast species composition by phytocenoses (**a**), and seasonality (**b**). NS: Neotropical savanna, IO: Iron outcrops, AF: Atlantic forest, and RA: Revegetated areas.

Four species showed a wide distribution, as they were recovered in three of the four phytocenoses (Figure 3a): *Candida parapsilosis*, *Schwanniomyces vanrijiae*, and *Meyerozyma guilliermondii* were found in the Atlantic forest, Iron outcrops, and the Revegetated areas. *Papiliotrema laurentii* was found in the Iron outcrops, Neotropical savanna, and Revegetated areas. The Atlantic forest and Revegetated areas shared the largest number of species: *Candida maltosa* (the most frequent species), *Wickerhamomyces anomalus*, *Saturnispora silvae*, *Candida parapsilosis*, *Schwanniomyces vanrijiae*, *Meyerozyma guilliermondii*, and *Cutaneotrichosporon terricola*. Seven species were recovered solely from the Revegetated area, including *Candida glabrata*, *C. quercitrusa*, and *Lachancea kluyveri,* and were represented by only one isolate.

### 3.4. Yeast Communities by Season

The yeast communities were similar in distribution between seasons in terms of species richness, with 23 taxa in dry winter and 24 in rainy summer (Table 3). However, the species composition differed between the observed seasons (Figure 3b). In rainy summer, *Candida maltosa* was the main species with the highest frequency of occurrence, followed by *Schwanniomyces vanrijiae*, *Rhodotorula mucilaginosa*, and *Meyerozyma guilliermondii*. The rainy summer had 14 unique species, including three red-pigmented species: *Rhodotorula dairenensis*, *R. mucilaginosa*, and *R. toruloides*. In dry winter, *C. maltosa* was the most frequent species, followed by *Papiliotrema laurentii* and *C. melibiosica*. The dry winter had 13 unique species, including *C. melibiosica* and *Hanseniaspora uvarum*. Ten species were found during both seasons (Figure 3b), including two of the most widely distributed species, namely *C. maltosa* and *P. laurentii*.

### 3.5. Multivariate Approach

**Figure 4:**
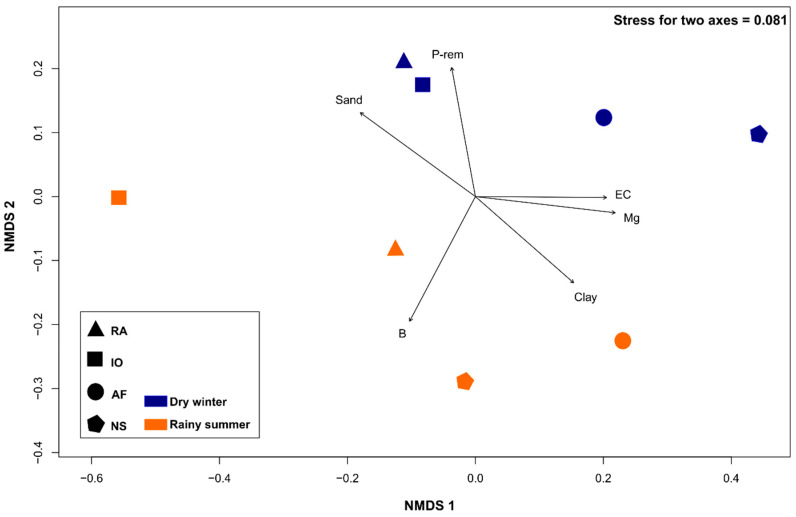
Nonmetric multidimensional scaling (NMDS) plot tracking community similarity by phytocenoses and seasonality. NS: Neotropical savanna, IO: Iron outcrops, AF: Atlantic forest, and RA: Revegetated areas. Soil variables (permutation test < 0.05)—Mg: magnesium, B: boron, P-rem: remaining phosphorus, EC: electrical conductivity, and texture (sand, clay).

The NMDS analysis showed a clear separation of yeast communities according to the four phytocenoses. The seasonality and soil attributes also influenced the distribution of yeast communities (Figure 4). The stress value (0.081) suggests that the data can be well represented in the 2-D ordination, preserving the original rank orders.

The micronutrients B, Mg, and P-rem, and the texture (sand and clay) were the variables with the most significant influence on the yeast community structure chosen by forward selection with the permutation test (P < 0.05). These variables explained 65% (P-rem and clay), 75% (Mg and B), and 77% (sand) of data variability. The yeast communities of the Iron outcrops and Revegetated areas seen in the rainy summer were the most similar, and positively correlated with the P-rem and sand content. The Iron outcrops area community in the dry winter was negatively correlated with the Mg content, and this ecosystem showed the lowest values for this attribute (Table 2). The clay content was positively correlated with the Atlantic forest area community, and the B content with the Neotropical savanna and Revegetated areas communities in the dry winter.

## 4. Discussion

The yeast community composition differed among the different phytocenoses in this study. Ascomycetous yeasts were more diverse in studied soils than basidiomycetous species, accounting for 29 and 8 species, respectively. We admit that this observation may reflect a bias in the used cultivation approach, because an enrichment step favors fermenting ascomycetous yeasts [38]. Despite the potential methodological bias, it is important to mention a few considerations. First, most of the available data on soil yeasts was obtained for boreal and temperate forests, which are generally species-poor and dominated by a dozen plant species [3,5,6]. Second, it has been demonstrated that plant communities influence soil yeast communities, e.g., grasslands and forests of different type, complexity, and heterogeneity [4]. Third, the high species diversity of plants results in high forest heterogeneity, as found in the studied Brazilian biomes. In our opinion, the high diversity of ascomycetous yeasts in the Atlantic forest and Neotropical savanna areas might reflect the high diversity and heterogeneity of the vegetation cover. Available results do not give the clear answer to the question of whether or not tropical soils in Brazil generally harbor smaller numbers of basidiomycetous yeasts. Our present study yielded soil-borne basidiomycetes, *Apiotrichum laibachii*, *Papiliotrema laurentii*, and *Saitozyma podzolica*. However, many other prominent soil-related types of yeast, like *Solicoccozyma* spp., have not been observed in this survey. It is important to study soils yeasts in other species-rich plant communities to understand whether the proportion of ascomycetous species is correlated with the great richness of vascular plants in tropical regions, including Brazil.

Thirty-seven yeast taxa were retrieved in this survey, which represents 86% and 66% of the diversity predicted by Chao2 and ICE species richness estimators, respectively (Figure 2). Our results showed that the sampling effort was sufficient to recover a fairly large proportion of the expected yeast diversity. Dissimilarities between the two predictions point to a peculiarity of the structure of soil yeast communities, namely the occurrence of frequent and rare species. The fact that the results of Chao2 estimator are lower than those of ICE indicates that a substantial proportion of species in the samples are singletons and doubletons, as noted in Table 3. This finding strengthen the fact that yeast species have an uneven distribution between samples [33], and are often detected as singletons and doubletons, in one or a few samples. Despite the aforementioned limitations of sampling and cultivation, it is important to note that our survey yielded more yeasts than previous studies in South America. Another study of yeasts in Neotropical savanna soils reported 23 yeast species [11]. In Argentina, temperate Patagonian forests yielded 18 to 28 soil yeasts [5,39]. Only one study in South America reported a higher diversity of yeasts (66 mycotoxin-producing species) from a large area of the Amazonian rainforest [9]. In Argentina, temperate Patagonian forests yielded 18 to 28 soil yeasts [5,39].

The yeast species richness was different among phytocenoses. Our results show greater species richness in the Revegetated area compared to natural ecosystems (Table 3). This finding confirms our hypothesis that the modification of the vegetation affects the composition of soil yeast communities, i.e., increased species richness in the Revegetated area following the secondary succession caused by mining activity (e.g., removal of native vegetation covers and revegetation with grasses). Higher species richness in the Revegetated area corroborates the results found for different functional groups of soil microorganisms in the iron-mining area of Brazil [13,20,21,22,23], including yeasts [24]. Our results are consistent with those reported for an area in the Brazilian Amazon rainforest, where the diversity of the soil microbial community increased after a deforestation event and forest conversion into permanent agricultural land [40]. Soil microbial communities tend to become more similar over time after the disturbance, resulting in the loss of beta-diversity [40]. Yeast communities in the Revegetated and Atlantic forest areas shared a large number of species. Since Atlantic forest area was the original vegetation before the disturbance event, these similarities may be indicating the resilience of indigenous species from the Atlantic forest biome. A high diversity of soil yeasts is expected in a biodiversity hotspot like the Atlantic forest. However, this was not the case of another native plant community, the Neotropical savanna area. We believe that lower yeast species diversity in the Neotropical savanna and Iron outcrops area is the result of unfavorable conditions for some species like high acidity, and heavy metal (aluminum and iron) content in soil conditions [12]. Nevertheless, these inhospitable soils yielded two potential new species (*Schwanniomyces* sp.1 and *Meyerozyma* sp.3), which will be further characterized by our team in more details. Overall, our results corroborate the importance of the vegetation cover as a driving force for soil yeast communities [5,13,24,41,42,43,44]. 

The frequency of occurrence of yeast species varied between phytocenoses. The most frequent yeast species in the present study, *Candida maltosa*, *C. melibiosica*, and *C. intermedia* (occurrence > 20%), are not commonly regarded as pedobiont species [38]. Species of the genus *Candida* were also the most frequent species detected in the Amazon rain forest [9], which is characterized by the dense vegetation and very humid climate. The frequency of fermentative yeasts (with a few exceptions ascomycetous) in tropical soils is believed to be related to predominantly hydromorphic and anoxic conditions, and the availability of simple sugars [45]. The most frequent species identified in this study was *Candida maltosa* in Atlantic forest (31%) and the Regevetated areas (60%). *Candida maltosa* has been reported as one of the most abundant in cultivated regions and soils under predominantly herbaceous vegetation [46,47,48]. *Candida melibiosica* was the most frequent species in the Iron outcrops area (43%), and *Candida intermedia*, the most frequent species in the Neotropical savanna area (20%). Other frequent species included *Schwanniomyces vanrijiae*, *Papiliotrema laurentii*, *Meyerozyma guilliermondii*, *Rhodotorula mucilaginosa*, and *Saitozyma podzolica*. These species are frequently reported from soils [9,49,50]. The yeast *S. vanrijiae* was frequent in the Atlantic forest area (25%). This species has often been associated with forest phytocenoses [4,11,38], including decaying wood in the Brazilian Atlantic Forest [51,52]. The yeast *P. laurentii* was frequent in the Iron outcrop (11%) and the Revegetated (11%) areas. *R. mucilaginosa*, which was frequent in the Neotropical savanna area (13%), is a common phylloplane yeast. Frequently found in the Neotropical savanna area (13%), *Saitozyma podzolica* is a typical soil-borne species inhabiting acid soils [53]. The distribution of this species has been strongly associated with aluminum content and acidity in Brazilian soils [11,24]. These abiotic soil parameters are characteristic for the Neotropical savanna area (Table 2). *Meyerozyma guilliermondii* is a transient species in soils. This yeast was among a few species that occurred in three phytocenoses: Iron outcrops (8%), Atlantic forest (2.7%), and the Revegetated areas (4.2%). 

Our results showed yeast community dynamics in response to a seasonal climate change. The seasonal dynamics affected species frequency, but not species richness. Among species detected in the rainy summer, we frequently noticed the group of pigmented (red) yeasts, *Rhodotorula toruloides* and *R. mucilaginosa*. These yeasts represent typical phylloplane species that are introduced to the soil habitat with falling leaves (or other plant material) and running waters [54]. Only in the rainy summer were we able to detect *Sugiyamaella xylolytica*, a recently described from rotting wood in Brazil new species in the *Sugiyamaella* clade [55]. The seasonal dynamics of yeast communities in soils has been so far demonstrated mainly for red-pigmented yeasts [3,56]. Distribution of other species and ecological groups did not show a clear trend. Like in our findings, red-pigmented *Rhodotorula* spp. were only detected during a rainy summer, demonstrating the input of yeasts with plant material and the effects of plants on the composition of soil communities. These findings reinforce the hypothesis of the entry of new species in the soil from plant debris during the rainy summer. These species originating from the deposition of plant residues are transient species quickly eliminated (overcompetitive or attacked) in the soil [57].

The NMDS analysis demonstrated the influence of the vegetation type, seasonality, and abiotic soil parameters on the structure of yeast communities. In addition, the analysis shows that soil characteristics, such as texture (sand and clay), nutrients (phosphorus), and microelements (Mg, B) significantly affected the composition of yeast communities. The texture (measured as proportions of clay and sand) explained most of the observed variability of yeast communities between the two seasons. Our statistical analysis showed a positive correlation between electrical conductivity and clay content (Figure 4). The relation between these two parameters has already been described, and it is believed that electrical conductivity reflects a combination of two parameters, soil content (moisture) and cation exchange capacity [58]. We believe that the influence of texture on the distribution of yeasts according in the two seasons is related to soil moisture (and the water retention capacity) and availability of nutrients for the microbial community. For example, a larger proportion of clay in the soil can lead to a higher availability of nutrients [59]. The availability of micronutrients Mg, B, and P-rem affected the composition of soil yeast communities in our study. The effect of micronutrients on the distribution of yeasts in the soil is not surprising, since these nutrients are essential for their growth and metabolism. Changes of yeast community parameters in relation to soil texture, moisture, temperature, and available nutrients have been demonstrated before [11,42,50,60,61]. However, it should be noted that soil parameters that significantly affect the distribution of soil yeasts are naturally connected, directly or indirectly, and positively or negatively, reducing their contribution in the observed statistical trend. 

The yeast species detected in this study have important ecological roles in the environment. For example, *Candida maltosa* has been shown to degrade pollutants such as hydrocarbons [48] and phenolic compounds [62], which indicates their involvement in the recovery of soils that have suffered from environmental damage. *Candida melibiosica* inhibited the growth of pathogenic fungi in culture [63], and *C. intermedia* is recognized for its capacity as a xylose-utilising yeast [64]. Some *P. laurentii* strains receive attention for laccase production [65]. These are enzymes associated with lignin transformation and phenolic compounds [66,67], suggesting a possible role for this species in lignin decomposition. *Rhodotorula mucilaginosa* has potential use as an indicator of environmental quality, mainly as a bio-indicator for heavy metal perturbations in low-nutrient soils [68]. Representatives of *M. guilliermondii* species have ecosystem-relevant traits, such as phosphate-solubilization [69,70], and provide insoluble nutrients in the soil microbial community [71]. These and other properties of soil yeasts point to a few possible ecological roles of these microscopic fungi in the ecosystem, including nutrient transformations, macronutrient solubilization (such as P and Ca, making them available to plants), synthesis of plant growth promoters (IAA), and antagonistic interactions with plant pathogens [2]. In South America, the importance and activity of yeasts in the decomposition of wood in Chilean rainforest was described [72,73]. Yeasts associated with wood decay are also described in the Amazonian rainforest, including several *Candida* species [51]. A larger taxonomic diversity of yeasts in the Revegetated area implies a larger diversity of traits and properties that can help to speed up the transformation and renaturation of the anthropogenically disturbed area. A detailed investigation of strains isolated in this study (own unpublished data) showed that they possess several traits, such as plant-growth promotion, through the production of indole acetic acid and phosphate solubilization. This observation strengthens the need for further analyses of yeast diversity and ecology for a better understanding of their roles in soil processes.

This study provided the first comprehensive inventory of soil yeasts in major phytocenoses of the Iron quadrangle region (Minas Gerais State, Brazil). Our results pointed to the importance of environmental parameters and vegetation as determinants of soil yeast community composition. Our findings also indicated a possible ecological role for yeast communities in the renaturation of anthropically disturbed areas, for example through a rapid colonization and alteration of available niches in soils. Yeast communities can be used as indicators of the quality of the environment [74]. Our results demonstrated that yeast communities in the Revegetated area are similar to those in soils under the natural vegetation, Atlantic forest community. This observation gives hope for future restoration of mining areas to a state similar to that of undisturbed habitats following a proper renaturation activity, like the one analyzed in the present study. Our results showed a positive effect of fast-growing grass species (*Urochloa brizantha* and *Panicum maximum*). These plants rapidly produce biomass that can be incorporated into the ecosystem and accelerate formation of the dense vegetation cover that prevents soil erosion.

## Figures and Tables

**Table 1:** Geographical characteristics of phytocenoses sampled in the Iron Quadrangle region, Minas Gerais, Brazil. NS: Neotropical savanna, IO: Iron outcrops, AF: Atlantic forest, and RA: Revegetated areas.

Geographical Characteristics	Phytocenoses			
	NS	IO	AF	RA
Locality	Nova Lima	Nova Lima	Brumadinho	Brumadinho
Latitude	20°6′42″S	20°6′52″S	20°9′4″S	20°9′35″S
Longitude	43°57′28″W	43°57′27″W	44°8′47″W	44°9′5″W
Annual Rainfall (mm)	1390	1390	1325	1325
Mean Annual Temperature (°C)	21.0	21.0	21.3	21.3
Impact level	None to low	None to low	None to low	High

**Table 2:** Mean values of the chemical and physical properties of the soils at different phytocenoses, in two climate seasons (winter and summer), in the Iron Quadrangle, Minas Gerais, Brazil.

Soil Physicochemical Parameters ^a^	Neotropical Savanna		Iron Outcrops		Atlantic Forest		Revegetated Area	
	Winter	Summer	Winter	Summer	Winter	Summer	Winter	Summer
pH (H_2_O)	4.97	4.66	4.72	4.46	4.21	3.91	5.6	5.36
K (mg dm^−3^)	72.6 a	83.8 a	56.8 a	64.0 a	75.6 a	78.0 a	88.2 a	85.2 a
P (mg dm^−3^)	1.363 b	1.623 b	1.595 b	2.275 b	2.151 b	2.125 b	1.66 b	3.626 a
Ca^2+^ (cmol_c_ dm^−3^)	0.91 a	1.15 a	1.28 a	1.476 a	0.99 a	1.146 a	0.75 a	1.154 a
Mg^2+^ (cmol_c_ dm^−3^)	0.38 a	0.461 a	0.24 a	0.269 a	0.45 a	0.506 a	0.3 a	0.364 a
Al^3+^ (cmol_c_ dm^−3^)	1.56 a	1.65 a	0.85 b	0.79 b	1.9 a	1.86 a	0.09 c	0.12 c
H+Al (cmol_c_ dm^−3^)	15.46 b	21.41 a	12.64 b	22.01 a	12.26 b	18.53 a	1.94 c	2.64 c
SB (cmol_c_ dm^−3^)	1.478 a	1.826 a	1.665 a	1.909 a	1.633 a	1.852 a	1.276 a	1.737 a
t (cmol_c_ dm^−3^)	3.03 b	3.47 a	2.51 b	2.69 b	3.53 a	3.71 a	1.36 c	1.85 c
T (cmol_c_ dm^−3^)	16.92 b	23.24 a	14.31 b	23.92 a	13.89 b	20.38 a	3.21 c	4.38 c
V (%)	12.72 b	10.79 b	13.56 b	10.86 b	13.64 b	11.17 b	40.73 a	40.10 a
m (%)	46.13 a	46.42 a	33.78 b	29.15 b	59.30 a	56.81 a	6.76 c	7.27 c
OM (g kg^−1^)	83.02 a	82.35 a	75.87 a	80.8 a	49.48 b	48.04 b	13.87 c	12.44 c
P-rem (mg/L)	4.56 d	7.65 d	12.61 c	20.10 a	11.02 c	16.51 b	11.03 c	20.10 a
Zn (mg dm^−3^)	3.136 a	3.129 a	3.292 a	3.443 a	1.927 b	1.687 b	1.608 b	2.137 b
Fe (mg dm^−3^)	134.53 b	149.05 b	403.73 a	401.78 a	124.79 b	136.04 b	150.81 b	85.32 b
Mn (mg dm^−3^)	112.29 a	107.40 a	88.88 a	72.45 b	40.76 b	45.62 b	103.99 a	86.93 a
Cu (mg dm^−3^)	0.799 b	0.293 b	0.57 b	0.357 b	0.809 b	0.561 b	2.14 a	1.917 a
B (mg dm^−3^)	0.202 a	0.122 b	0.266 a	0.123 b	0.201 a	0.102 b	0.154 b	0.106 b
S (mg dm^−3^)	36.29 b	21.33 d	26.58 c	15.02 d	29.06 b	20.19 d	45.14 a	31.08 b
Clay (g kg^−1^)	376 b	362 b	214 c	193 c	456 a	478 a	249 c	240 c
Silt (g kg^−1^)	243 a	275 a	174 b	183 b	188 b	178 b	262 a	277 a
Sand (g kg^−1^)	381 c	363 c	612 a	624 a	356 c	344 c	489 b	483 b
EC (mS/cm)	0.061 b	0.21 a	0.052 b	0.057 b	0.228 a	0.182 a	0.027 b	0.088 b

^a^ Means followed by the same letter do not differ from each other. The Scott–Knott test was applied at 5% probability. K: Potassium, P: Phosphorus, Ca: Calcium, Mg: Magnesium, Al: Aluminum, H + Al: total acidity, SB: sum of exchangeable bases, t: effective cation exchange capacity, T: cation exchange capacity at pH 7, V: saturation index of bases, m: saturation index of aluminum, OM: organic matter, P-rem: remaining phosphorus, Zn: Zinc, Fe: Iron, Mn: Manganese, Cu: Copper, B: Boron, S: Sulfur, and EC: electrical conductivity.

**Table 3:** Occurrence frequency of yeasts in soils from different phytocenoses, in two distinct seasons. S(obs): observed species richness, si: singletons, do: doubletons. NS: Neotropical savanna, IO: Iron outcrops, AF: Atlantic forest, and RA: Revegetated areas.

Yeast Species	GenBank Reference	Frequency (%)					
		Phytocenoses				Seasonality	
		NS	IO	AF	RA	Winter	Summer
Ascomycota							
Saccharomycetales							
*Candida maltosa*	KJ159031			31	60	43.70	40.00
*Candida melibiosica*	KY106567		43			11.85	
*Candida parapsilosis*	MG871743		11	si	2.4		5.63
*Candida pseudolambica*	KY106707			5.3		2.96	
*Candida neerlandica*	NG054776			do			Do
*Candida sanyaensis*	NG054829			si			Si
*Candida intermedia*	MG815863	20			si	2.96	
*Diutina rugosa*	HE716783	do				do	
*Candida edaphicus*	AB247371	si		si			Do
*Candida insectorum*	JN544058		si			si	
*Candida quercitrusa*	KY996729				si	si	
*Candida glabrata*	MG859667				si		Si
*Candida* sp. (Kurtzmaniella clade)	MG833304		si	do			1.88
*Schwanniomyces vanrijiae*	NG054865		si	25	si	do	11.88
*Schwanniomyces polymorphus*	KY109623	do		do		2.96	
*Schwanniomyces* sp.1	LN909492		si				Si
*Debaryomyces* sp.2	NG055699	si			si	do	
*Meyerozyma guilliermondii*	KY952849		8	do	4.2	si	6.88
*Meyerozyma* sp.3	KY952849		si		1.8	2.22	Si
*Barnettozyma californica*	MG707647			6.7		do	1.88
*Hanseniaspora uvarum*	KP990659				3	3.70	
*Pichia kudriavzevii*	MF769603				2.4	2.22	Si
*Kodamaea ohmeri*	KY684049	si	do			si	Do
*Wickerhamomyces anomalus*	MF769591			do	si	si	Do
*Torulaspora delbrueckii*	MH010872			si			Si
*Torulaspora* sp.4	KY109871				1.8		1.88
*Sugiyamaella xylolytica*	KF889433			do			Do
*Saturnispora silvae*	MG707692			si	si		Do
*Lachancea kluyveri*	KY108237				si	si	
**Basidiomycota**							
Tremellales							
*Papiliotrema laurentii*	KY037816	si	11		11	12.59	4.38
*Saitozyma podzolica*	KX903051	do		8		do	3.75
*Apiotrichum laibachii*	JQ672597		si			si	
*Apiotrichum loubieri*	KY106137		si			si	
*Cutaneotrichosporon terricola*	FJ527226			si	si	do	
Sporidiobolales							
*Rhodotorula mucilaginosa*	KY744132	do			6		7.50
*Rhodotorula toruloides*	KF971831		si		do		1.88
*Rhodotorula dairenensis*	KY108994				do		Do
**S (obs)**		9	13	17	20	23	24
**Singletons (si)**		4	8	6	9	8	6
**Doubletons (do)**		4	1	6	2	6	7
